# Rapid high resolution T1 mapping as a marker of brain development: Normative ranges in key regions of interest

**DOI:** 10.1371/journal.pone.0198250

**Published:** 2018-06-14

**Authors:** Sylvain Eminian, Steven David Hajdu, Reto Antoine Meuli, Philippe Maeder, Patric Hagmann

**Affiliations:** Department of Diagnostic and Interventional Radiology, University of Lausanne and Lausanne University Hospital (UNIL-CHUV), Lausanne, Vaud, Switzerland; University of California Los Angeles, UNITED STATES

## Abstract

**Objectives:**

We studied in a clinical setting the age dependent T1 relaxation time as a marker of normal late brain maturation and compared it to conventional techniques, namely the apparent diffusion coefficient (ADC).

**Materials and methods:**

Forty-two healthy subjects ranging from ages 1 year to 20 years were included in our study. T1 brain maps in which the intensity of each pixel corresponded to T1 relaxation times were generated based on MR imaging data acquired using a MP2RAGE sequence. During the same session, diffusion tensor imaging data was collected. T1 relaxation times and ADC in white matter and grey matter were measured in seven clinically relevant regions of interest and were correlated to subjects’ age.

**Results:**

In the basal ganglia, there was a small, yet significant, decrease in T1 relaxation time (-0.45 ≤R≤-0.59, p<10^−2^) and ADC (-0.60≤R≤-0.65, p<10^−4^) as a function of age. In the frontal and parietal white matter, there was a significant decrease in T1 relaxation time (-0.62≤R≤-0.68, p<10^−4^) and ADC (-0.81≤R≤-0.85, p<10^−4^) as a function of age. T1 relaxation time changes in the corpus callosum and internal capsule were less relevant for this age range. There was no significant difference between the correlation of T1 relaxation time and ADC with respect to age (p-value = 0.39). The correlation between T1 relaxation and ADC is strong in the white matter but only moderate in basal ganglia over this age period.

**Conclusions:**

T1 relaxation time is a marker of brain maturation or myelination during late brain development. Between the age of 1 and 20 years, T1 relaxation time decreases as a function of age in the white matter and basal ganglia. The greatest changes occur in frontal and parietal white matter. These regions are known to mature in the final stage of development and are mainly composed of association circuits. Age-correlation is not significantly different between T1 relaxation time and ADC. Therefore, T1 relaxation time does not appear to be a superior marker of brain maturation than ADC but may be considered as complementary owing the intrinsic differences in bio-physical sensitivity. This work may serve as normative ranges in clinical imaging routines.

## Introduction

Normal brain maturation from fetal life to the third decade of adulthood is denoted by structural and morphological changes visualized as progressive myelination, increase in brain size, and accordingly changes in MRI contrast [1,2]. From the last weeks of gestation to the first post-natal months, these changes are particularly dramatic [3]. Important structural remodeling occurs such as neuronal and glial migration as well as differentiation [4,5]. Later, changes during childhood and adolescence are characterized by axonal pruning, myelination of White (WM) and Gray matter (GM) and volume expansion. These patterns are regionally asynchronous with primary sensory and motor cortices occurring before secondary sensory, multisensory, associative and prefrontal cortices. Cerebral development progressively slows and is completed with the development of the prefrontal areas signaling full maturity in the late twenties [6]. With the advent of MRI, these phenomena can be investigated *in vivo* and non-invasively [7,8].

Paus reviews three maturational stages using qualitative patterns of T1 and T2-weighted MR images [9]. In the first or infantile stage (0–6months), the GM and WM contrast pattern is the opposite to that of a normal adult. WM intensity is lower than GM on T1-weighted images, and conversely higher in T2-weighted images. The second or “iso-intense pattern” stage (8–12 months) is characterized by weak contrast between GM and WM. Finally, the third or “the early-adult pattern” stage (>12 months) is characterized by higher WM intensity on T1-weighted images compared to GM intensity. T2-weighted signal is lower in the WM than in the GM [9]. These image-contrast changes, albeit qualitative, are observational, and subjective. Precise and dynamic maturational staging of an individual subject is not obtainable with this staging system. To overcome these limitations, an alternative using MRI-based quantitative staging is warranted.

Available magnetic resonance techniques to quantitatively measure brain maturation are T1 mapping, T2 mapping, quantitative Magnetization Transfer (qMT) and diffusion-based Fractional Anisotropy (FA) and Apparent Diffusion Coefficient (ADC). As we describe in detail later in the introduction, diffusion imaging has been studied extensively in this context (e.g. [10] while other quantitative imaging techniques such as T1 and T2 relaxometry are only starting to emerge in the clinical context thanks to the development of relatively rapid and robust imaging sequences. T1 and T2 relaxation properties depend narrowly on the concentration of macro-molecules such as proteins, phospholipids polysaccharids and fat; as wells as on the level of binding of water to these macro-molecules. Schematically, bound protons (protons of macromolecules and water protons bound in the vincinity to macromolecules) have very short T2 and T1 [11]. Hence brain maturation, which goes along with increased concentration of myelin, is characterized by T1 and T2 shortening [12].

T1 relaxation time can be imaged with various T1 mapping techniques, such as precise and accurate inversion-recovery (PAIR) [13,14] or driven equilibrium single pulse observation of T1 (DESPOT1) [15]. Alternatively, T2 relaxation time can be measured with T2 mapping techniques as reported in by Ding et al. [16] or Deoni et al. [15]. Quantitative Magnetization Transfer imaging is a technique based on the exchange of proton magnetization between water molecules and macromolecules allowing the visualization of changes in macromolecular tissue composition [17]. The primary source of magnetization transfer change in WM is the lipid-rich myelin. Diffusion Tensor Imaging (DTI) is based on Brownian motion of water as it diffuses through the brain [18]. The standard tissue diffusion parameters are the ADC and the FA. ADC reflects the restriction of water molecule motion due to the density of obstacles such as myelin, cell membranes and macromolecules whereas FA is a measure of the directionality of diffusion, correlating to fiber tract orientation. Both are sensitive to myelin content [19,20]. All the above mentionned techniques are limited by the absence of absolute myelin quantification, as they all measure the interplay among complex biological processes where maturation in general and myelination in particular play a role.

T1 relaxation time decreases as a function of age, significantly in the first three months of life and later levelling off during adolescence, related to brain maturation and myelination, as demonstrated by older studies [13,21,22]. Different decreasing rates were noted for the basal ganglia and WM with regional dependence. However, establishing a normal range of T1 relaxation times in relation to age is challenging owing to the heterogeneity of age groups and the small number of subjects for analysis. Nevertheless, using new and efficient quantitative T1 relaxation time imaging Schneider et al showed important quadratic changes as a function of gestational age [23], while Deoni and colleagues reported in large populations logarithmic changes in T1 relaxation in the WM not only in infants [24] but also over an age range from 3 months to 5 years of age [25] and in the cortex in a similar age range [26]. Finally a lifespan study of WM maturation and degeneration using T1 mapping was performed by Yeatman et al [27].

Magnetization-prepared two rapid acquisition gradient echos (MP2RAGE) is an imaging sequence that has initially been designed to overcome large spatial inhomogeneity in the B1 magnetic field seen at high static B0 magnetic fields [28]. To overcome this effect, the T1-weight magnetization-prepared rapid gradient echo (MPRAGE) sequence was modified to generate two different images at different inversion times. The correlate is that T1 relaxation time can be estimated from those two images.

Numerous studies correlating ADC to age in children and adolescent were performed and show a similar characteristic quantitative decreasing curve as T1 relaxation [10,29–36]. They show also various regional trends. Watanabe and colleagues observed in 138 patients, two distinctive maturation periods [10]. During the first period, between 0–2 years of age, ADC decreases rapidly and logarithmically as a function of age. During the second period, between 2–20 years, ADC further decreases logarithmically but at a slower rate. Löbel and colleagues described similarly a logarithmic decrease in each region of interest in a population of 72 patients ranging from three weeks to 19 years of age [29]. Engelbrecht and colleagues observed in sample of 44 children with an age range between 7 days to 7.5 years, a monoexponential decrease for all anatomic regions [7]. The observed ADC decreases as a function of age and corresponds to water loss [37] wrapping of axons by the oligodendroglial process [38] and myelination, increasing macromolecular concentration, membrane surface-to-cell volume ratio and axonal diameters [39]. In adulthood (18–84 years) diffusion related metrics change linearly, as shown by a study by Arshad et al [40] who also noticed that diffusion parameters behave differently from Myelin Water Fraction.

In the light of previous work the aim of the present study is to report normative T1 relaxation time in the brain of children ranging from 1 to 18 year old, using a clinically usable, readily available and FDA approved sequence, namely MP2RAGE (Siemens Medical, Erlangen, Germany). A region of interest (ROI) approach has been chosen in order to be close to clinical practice where automatic or manual segmentation of brain structures is not necessarily performed routinely. This work should help clinician characterizing their patients’ maturation level.

## Materials and methods

Patients, ranging in age from 1 and 20 years, investigated for either headache or suspected epileptic seizure and who underwent cerebral MR studies with or without sedation using a MP2RAGE sequence at 3.0 Tesla between May 2013 to December 2014 were identified from a retrospective review of medical records. A total of 200 patients were selected as having no neurological or psychiatric abnormalities and no brain lesions or structural defects. A total of 42 patients (16 males, 26 females) were recognized as having normal imaging findings and no significant medical history and were included in our study. The cohort was made of 16 males and 26 females without significant age difference (p = 0.5786). Among these patients, 36 exams included an additional DTI sequence and were used in our comparative analysis. The study protocol 454/14 was approved by the local institutional review board (Commission cantonale (CV) d’éthique de la recherche sur l’être humain (CER-VD)) on 27 January 2015.

### MRI protocol for the T1 maps and diffusion

Three different 3.0 Tesla MR systems (MAGNETOM Trio, Verio, and Skyra; Siemens AG, Healthcare, Erlangen, Germany) using a 32-channel head coil were used. The MP2RAGE sequence [28] is an isotropic gradient echo sequence, free from B1-inhomogeniety, with two inversion pulses generating two images acquired at two separate inversion times (TI_1_ and TI_2_) which are subsequently combined using the equation:

$$MP2RAGE=\frac{{GRE}_{TI1}\;{GRE}_{TI2}}{{GRE}_{TI1}^{2}+{GRE}_{TI2}^{2}}.$$

The particularity of the sequence is the low flip angle gradient with a short TR. The sequence was programmed with the following parameters: repetition time (TR) = 5000, echo time (TE) = 2.94, first inversion time (TI_1_) = 700 ms, second inversion time (TI_2_) = 2500 ms, 160 slices, matrix = 256 x 256 points, field of view = 256 x 256 mm^2^ yielding a voxel size of 1 x 1 x 1.2 mm. Scan time was 8 minutes and 22 seconds. The exact acquisition parameters are provided in the supplementary material (S1 File). The MPRAGE sequence is FDA approved for clinical use and in this context a validation study on the accuracy of the T1 relaxation measurements has been conducted (S2 File). Diffusion imaging was performed using a standard spin echo sequence with echo planar read-out (EPI) in 6 diffusion encoding directions. The sequence was programmed with the following parameters: TR = 6600 ms, TE = 95 ms, b value = 0 and 1000[s/mm^2^] averaged 5 times, 43 slices, matrix size = 156 x 156 points, field of view = 218 x 218 mm^2^ and a slice thickness of 3.3 mm, yielding in plane resolution of 1.4 x 1.4 mm^2^. Scan time was 4 minutes and 26 seconds. ADC maps are automatically computed by the scanner as the summe of the diagonal of the diffusion tensor matrix divided by 3.

### Regions of interest analysis

**Fig 1:**
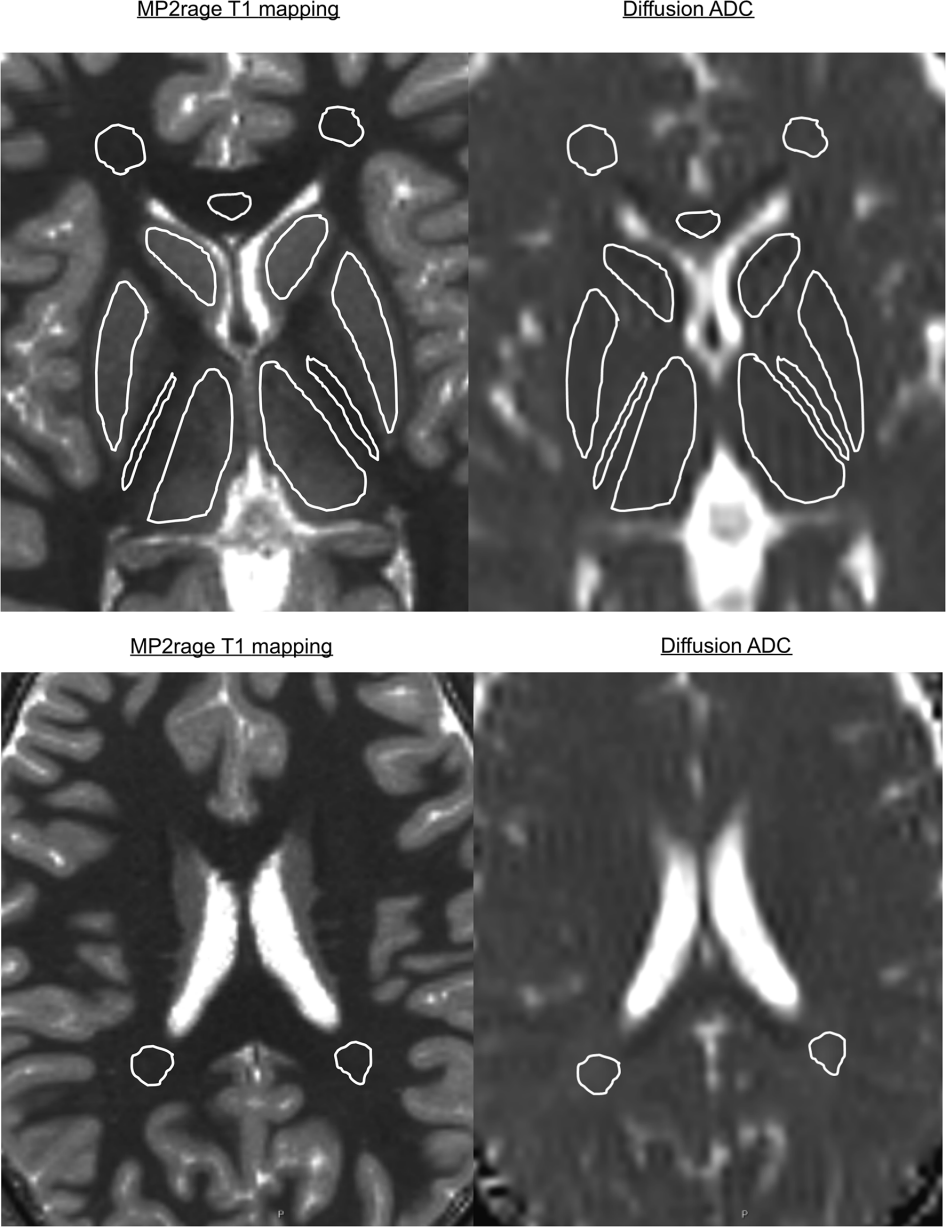
Region of interest analysis on the T1 and ADC maps. This was performed by placing ROIs in six areas: thalamus, posterior limb of the internal capsule, putamen, caudate nucleus, genu of the corpus callosum, and frontal white matter in an axial slice parallel to the bi-commissural plane as shown. Another ROI in the parietal white matter was traced in a different axial slice.

A region of interest (ROI) analysis on the T1 and ADC maps was performed by placing ROIs in seven different anatomical regions: thalamus, posterior limb of the internal capsule, putamen, caudate nucleus, genu of the corpus callosum, parietal and frontal lobe WM(Fig 1). The ROIs were traced manually to minimize the partial volume effect. The ROIs were chosen in a plane parallel to the bi-commissural plane passing through the striatum for all the ROIs except for the parietal WM, which was traced on a separate slice. The collected values are provided in a spreadsheet format as supplementary material (S1 Table). Upon request, the original images are available.

### Data analysis

Age-related changes in T1 relaxation time and ADC were studied using Matlab (MathWorks, Massachusetts, United States). Pearson correlation coefficients (R) and associated p-values (p) were calculated for the logarithmic transformations (log(.)) of T1 relaxation times and ADC as a function of age for each ROI. T1 relaxation times were plotted in logarithmic scale for each ROI (mean value inside ROI) as a function of age on graphs containing their regression line with 95% confidence interval for each ROI. Similarly, ADC values were graphically represented. In supplementary information we provide similar analysis without logarithmic transformation (S3 File).

In order to check for gender effect, 16 male subjects were matched with 16 female subject of identical age. For each region, a Student’s t-test was performed to search for potential significant gender difference (S3 File).

T1-relaxation times of individual ROIs were correlated to the corresponding ADC values. Two scatter plots containing linear regression and 95% confidence interval were generated to graphically demonstrate the T1-ADC correlation for the three GM regions and four WM regions.

Finally, the distribution of regional correlation coefficients of T1-relaxation time and ADC were compared using a Student’s t-test.

## Results

**Fig 2:**
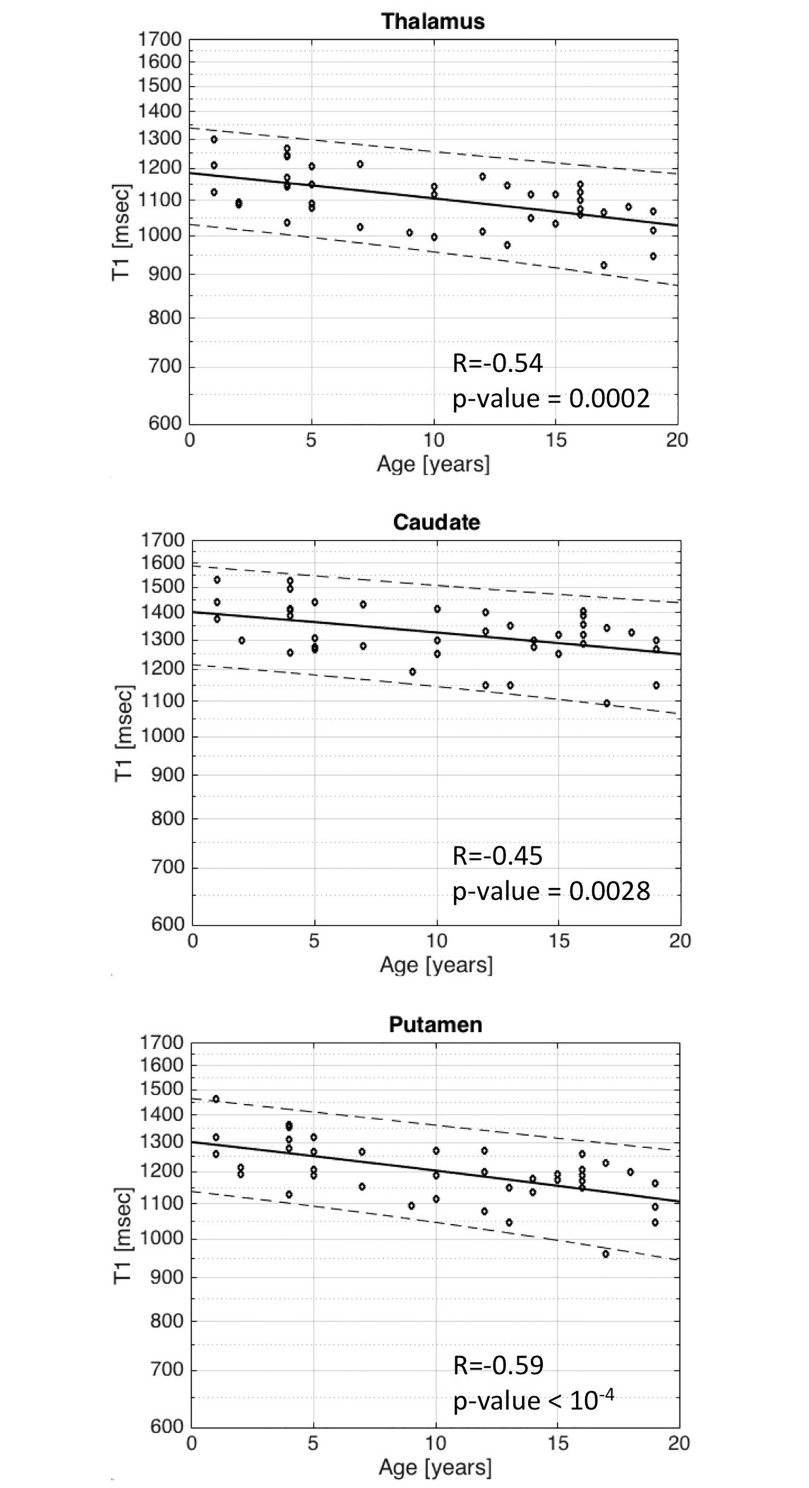
Scatter plots with superimposed linear regression lines correlating logarithm of T1 relaxation time in the basal ganglia with age. a. Thalamus b. Caudate c. Putamen. There is a slight, yet significant, decrease in T1 relaxation time with respect to age. They are accurately for Thalamus R = -0.54/p-value = 0.0002, for Caudate R = -0.45/p = 0.0028 and for Putamen R = -0.59/p<10^−4^.

**Fig 3:**
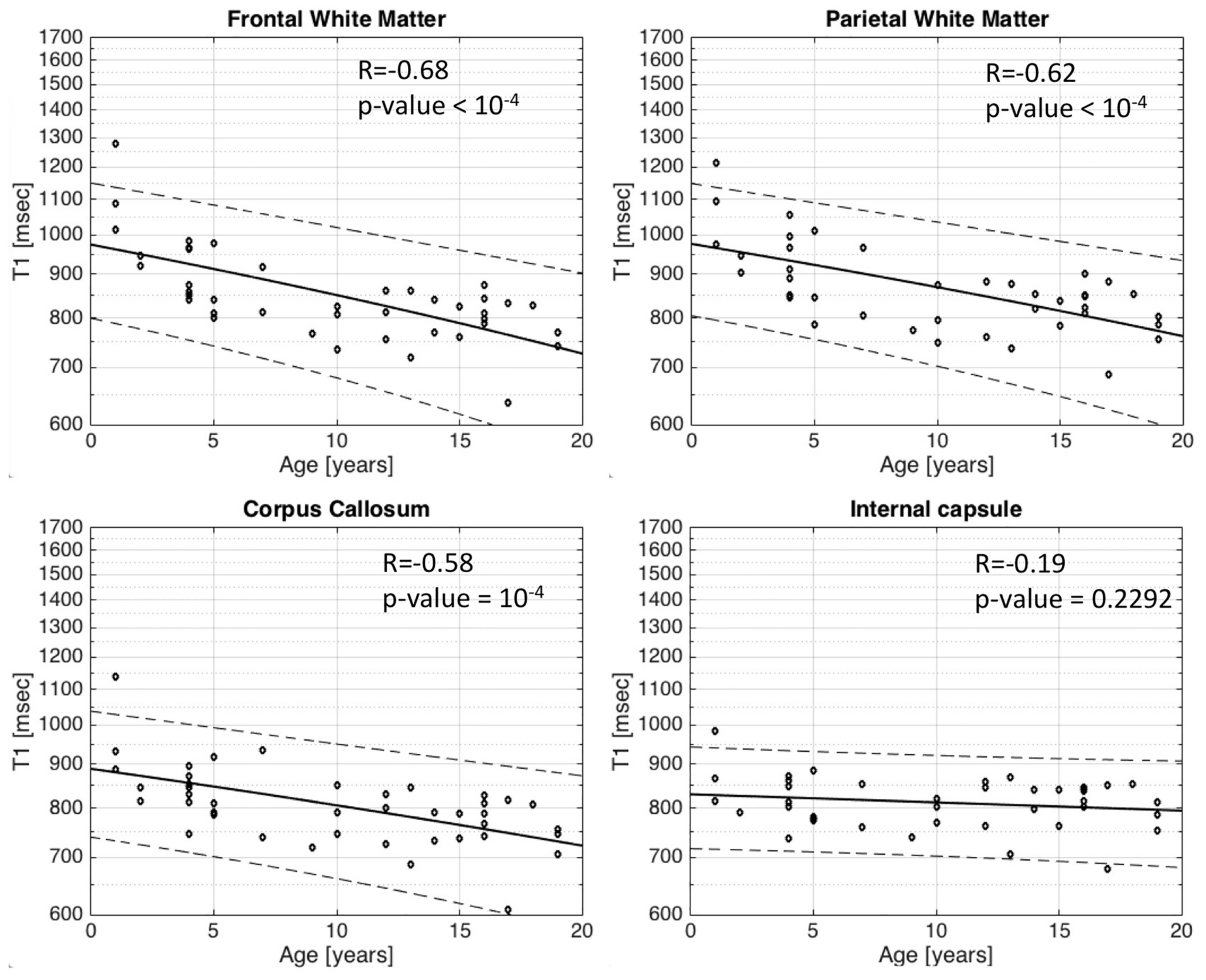
Scatter plots with superimposed linear regression lines correlating logarithm of T1 relaxation time in several white matter locations with age. a. Frontal white matter b. Parietal white matter c. Corpus callosum d. Internal capsule. The change of T1 relaxation with age is greater in the white matter with a p-value of <10^−4^ for Frontal white matter (R = -0.68), Parietal white matter (R = -0.62) and Corpus callosum (R = -0.58). This trend was not observed in the internal capsule with a R = -0.19/p-value = 0.23, most probably related to the fact that the internal capsule is one of the first structure to myelinate around birth.

Regarding T1 relaxation time in the basal ganglia, there is a slight, yet statistically significant, decrease in T1 relaxation time with respect to age within the thalamus R = -0.54/p-value = 0.0002 (Fig 2a), caudate nucleus R = -0.45/p = 0.0028 (Fig 2b) and putamen R = -0.59/p<10^−4^ (Fig 2c). More importantly, T1 relaxation times in three out of four WM regions studied decrease more significantly (p = <10^−4^) with age, in the frontal lobe (R = -0.68) (Fig 3a), parietal lobe WM (R = -0.62) (Fig 3b) and corpus callosum (R = -0.58) (Fig 3c). The fourth WM region studied, the posterior limb of the internal capsule, did not show any correlation between T1 relaxation time with respect to age (R = -0.19/p-value = 0.23) (Fig 3d).

**Fig 4:**
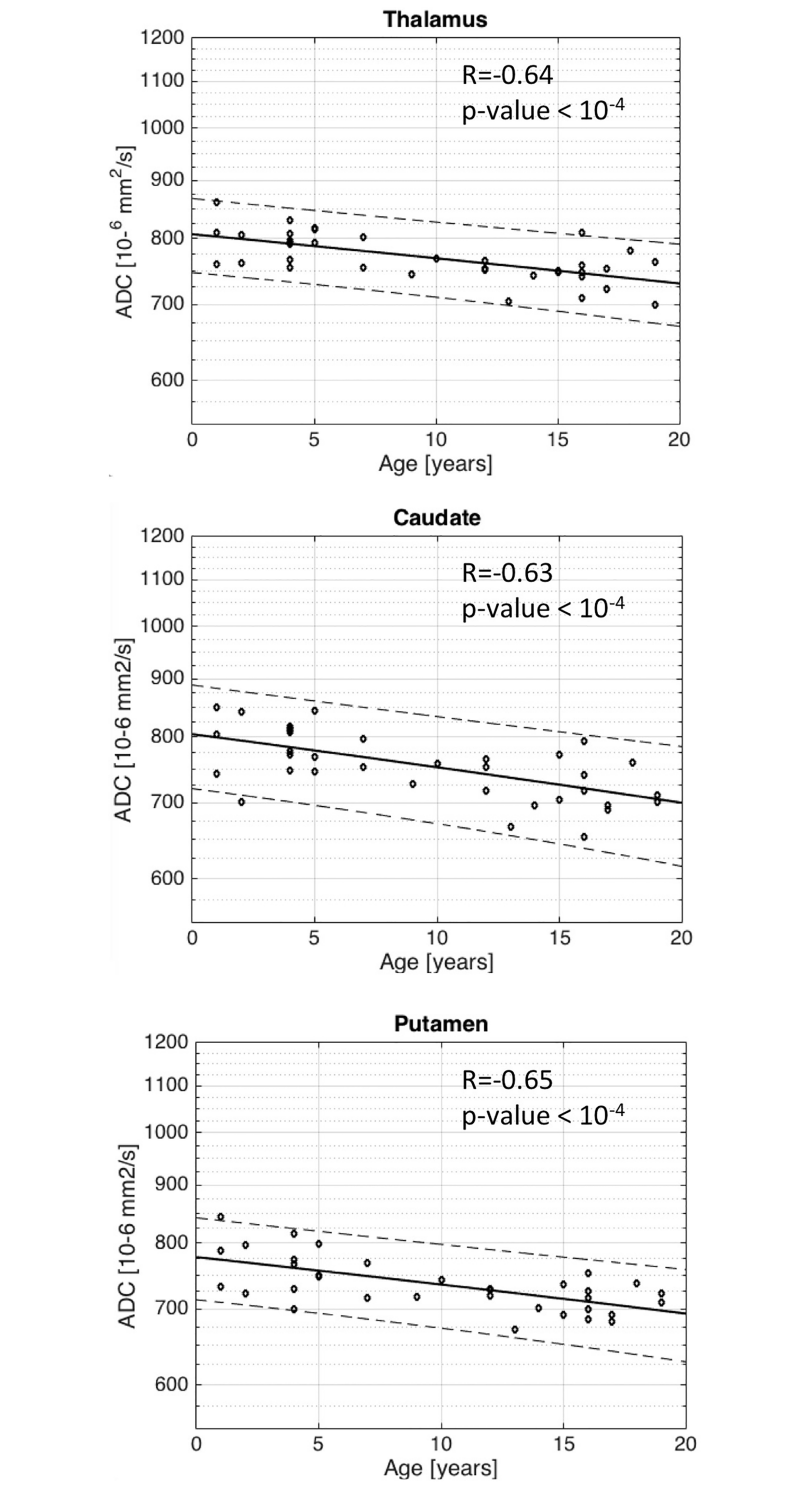
Scatter plots with superimposed linear regression lines correlating logarithm of ADC with age in the basal ganglia. a. Thalamus b. Caudate c. Putamen. There is a strong (R = -0.64 for Thalamus, R = -0.63 for Caudate, R = -0.65 for Putamen) and significant (with a p-value <10^−4^) decrease in ADC with respect to age.

**Fig 5:**
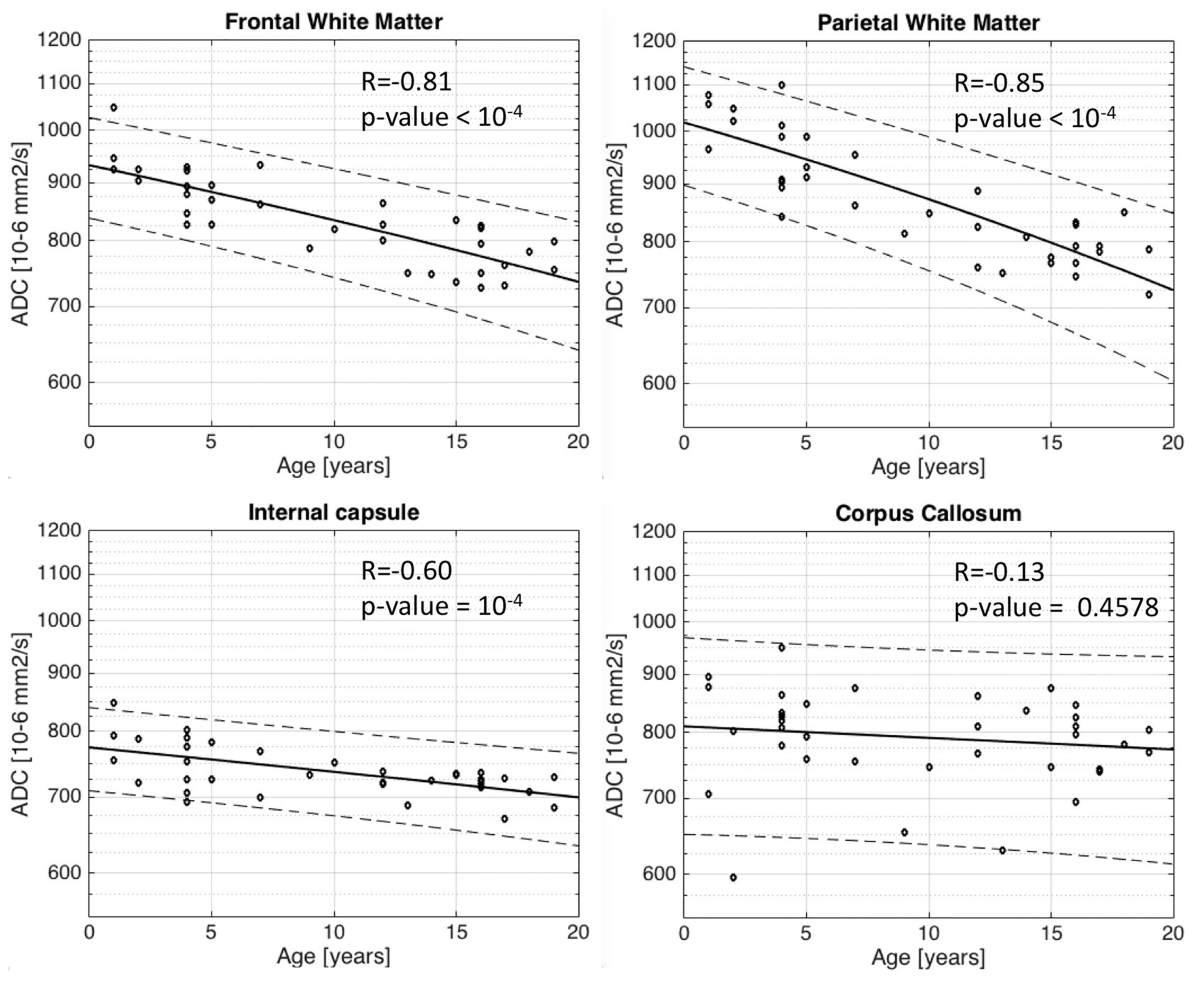
Scatter plots with superimposed linear regression lines correlating logarithm of ADC with age for white matter. a. Frontal white matter b. Parietal white matter c. Corpus callosum d. Internal capsule. The change of ADC with age is significant (p-value <10^−4^) and stronger in the white matter for Frontal white matter(R = -0.81), Parietal white matter(R = -0.85) and Internal Capsule(R = -0.60). The notable exception is for the Corpus callosum with R = -0.13/p-value = 0.45. This is probably due to the fact that there is a partial volume with the ventricle for this region of interest on the ADC map.

Logarithm of ADC decrease correlates strongly with age in the basal ganglia (p <10^−4^). Strong correlation is observed (R = -0.64) in the thalamus (Fig 4a), in the caudate nucleus (R = -0.63) (Fig 4b), and in the putamen (R = -0.65) (Fig 4c). A similar relationship is observed for ADC in the white matter as a function of age, determined as significant (p <10^−4^) in the frontal lobe (R = -0.81) (Fig 5a), in the parietal lobe (R = -0.85) (Fig 5b) and the posterior limb of the internal capsule (R = -0.60) (Fig 5c). Interestingly, no age correlation to ADC is observed in the corpus callosum (R = -0.13/p-value = 0.45) (Fig 5d).

We observed no gender effect in any ROIs, neither on T1 relaxation time nor on ADC (see supplementary material, S3 File).

**Fig 6:**
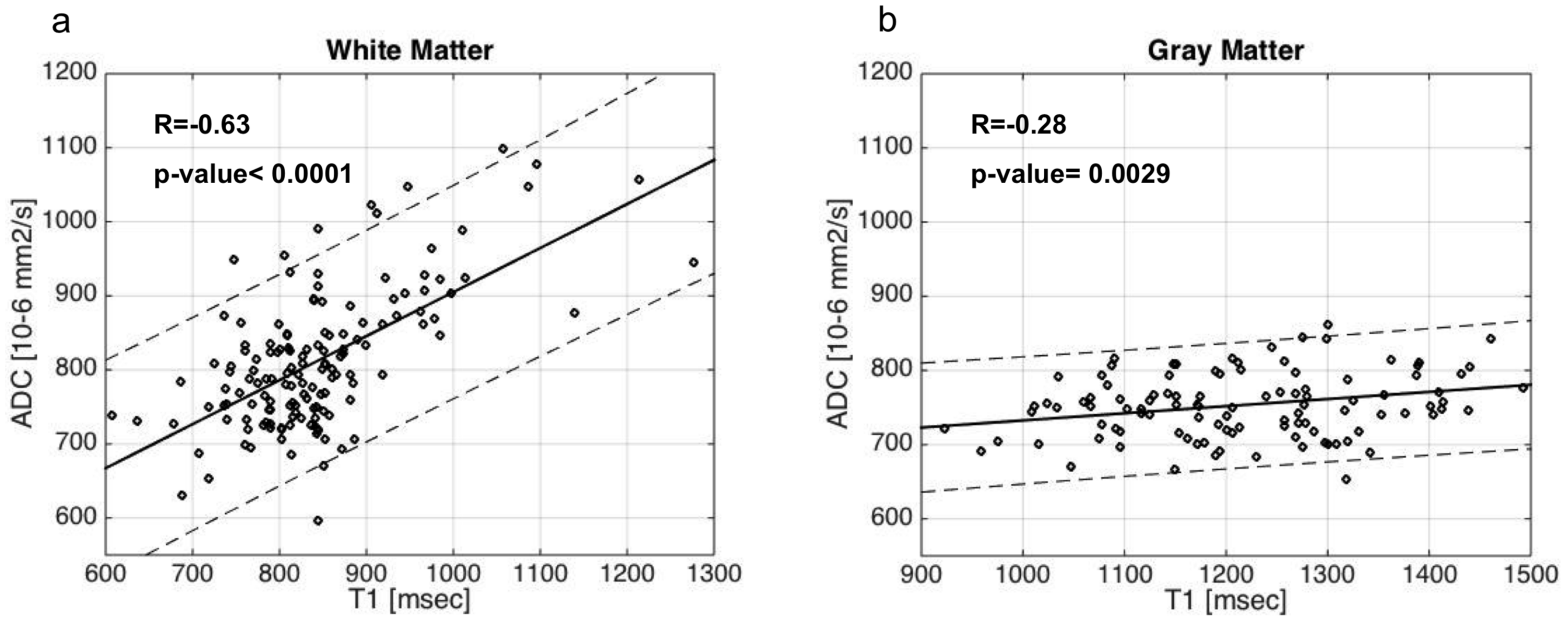
Correlation ADC/T1. a. WM b. GM. If we separate white matter from basal ganglia, the between the two contrasts is strong in the white matter (R = -0.63/p-value <0.0001) but still moderate in basal ganglia (R = -0.28 /p-value = 0.0029).

When grouping WM and GM regions separately, correlations between ADC and T1-relaxation time is strong in the WM (R = -0.63/p-value <0.0001) (Fig 6a) and moderate in basal ganglia (R = -0.28 /p-value = 0.0029) (Fig 6b).

The current theory is that T1 relaxation changes as a function of the concentration of macromolecules (i.e. myelin concentration) through spin-lattice energy dissipation [41,42]. Water mobility (i.e. ADC) changes not only with myelination level but also with the tortuosity of the environment [43,44].

In order to explore the difference in sensitivity to brain maturation between T1 relaxation time and ADC, the distribution of brain structure correlation coefficients (R) for T1-relaxation time was compared the distribution of correlation coefficients (R) for ADC (Table 1). No significant difference between the correlation of T1 relaxation and ADC was observed (p-value = 0.39).

**Table 1:** Comparing correlation coefficients (R) using MPRAGE vs ADC.

	MP2RAGE (N = 42)	ADC (N = 36)
Thalamus	-0.54	-0.64
Internal Capsule	-0.19	-0.6
Putamen	-0.59	-0.65
Caudate	-0.45	-0.63
Frontal White Matter	-0.68	-0.81
Corpus Callosum	-0.58	-0.13
Parietal White Matter	-0.62	-0.85
Average	-0.52	-0.62
	p-valeur	0.39

There is no significant difference in the age dependence between T1 relaxation time and ADC (p = 0.39).

## Discussion

Magnetic resonance imaging has been applied in measuring the development of the human brain. Along with the micro-architectural changes, myelination serves as an important marker of brain maturation and MRI sequences sensitive to minute changes should be preferentially used. Currently, quantitative measurement of brain maturation includes T1 and T2 mapping, qMT and FA, ADC. Nowadays time efficient imaging techniques for T1 mapping are becoming available [28,45]. MP2RAGE is an efficient T1 mapping technique that allows collecting images at milimeter resolution in clinically reasonable time (8 min) and provides simultaneously an unbiased T1 weighted high resolution anatomical image making additional diagnostic T1 weighted imaging unnecessary. We took a ROI approach because we wanted a simple an practical method easily applicable in all clinical settings where automatic and detail segmentation of different brain structures is not necessarily done. We have chosen regions in the WM and GM that are easy to identify and sufficiently large to be measurable with manually placed ROIs.

T1 relaxation time is an appropriate method for mapping myelin concentration as shown by Stüber et al [12], who correlated post-mortem human brain tissue T1 and T2* relaxometry with mapping of iron and phosphorus using proton induced X-ray emission. Indeed, myelin represents a large amount of brain volume and is made of hundreds of different proteins and lipids contributing to changes in T1 relaxation. Henceforth T1 relaxation can be considered as a biomarker of brain maturation.

In examining the WM, we observe a weak T1-age correlation in the posterior limb of the internal capsule with the lowest average extrapolated T1-relaxation time at birth of around 820 msec remaining approximately constant as a function of age, owing the known precocious development, i.e. myelination, of projection fibers of this region [1]. Commisural fibers, represented by the corpus callosum, show initially slightly higher T1-relaxation times at birth around 900 msec in addition to strong correlations in T1-relaxation time decrease with age because of the natural progression of maturation. The deep frontal and parietal WM regions are known to mature latest, hence the highest initial T1 relaxation time at around 1000 msec. Similarly, Steen and colleagues [13] reported a statistically significant correlation with R^2^ = 0.73 in frontal WM decrease as a function of age in tested subjects aged between 4 and 30 years.

Initial T1 relaxation time in the deep gray nuclei is higher than in the WM as expected since their myelin concentration is lower. Correlation of T1-relaxation time with subject age is less apparent in the caudate nucleus, putamen and thalamus relative to the aforementioned WM regions, but nonetheless significant, owing to a progressive, but less considerable myelination during post-natal maturation corresponding with the previously reported maturation studies using diffusion studies with FA [46].

It is known since the late nineties, and the works by Beaulieu et al (see [47] for review), that an important determinant of water restriction are cellular membrane and myelin concentration. However, in contrast to T1 relaxation, fiber orientation and dispersion, axonal diameter also play an important role in the ADC of brain tissue.

In concordance with its existing use in mapping brain maturation, ADC inversely correlates strongly and significantly as a function of age in our study. This is observed also in five out of six studied regions already described by Watanabbe and colleagues [29], Löbel and colleagues [32] and Engelbrecht and colleagues [7]. In our study the corpus callosum was the only region without significant correlation. The significant amount of partial volume averaging with the ventricles is the most plausible explanation of the confounded ADC maps.

In our study, no significant difference between the distribution of T1-relaxation and ADC correlation coefficients with respect to age was observed. Therefore, in our hands, T1 relaxation does not seem to correlate more with brain maturation than ADC. The correlation between ADC and T1 relaxation is significant, yet not very strong. This may point to the fact that those two contrasts, which both relate to maturation, reflect different biological processes. This hypothesis is also supported by the work of Arshad et al [40] who noted differences in DTI measures as compared to T2 derived Myelin Water Fraction (MWF) imaging and showed that while DTI measures exhibit a linear change between 18 and 84 years old, MWF exhibits a quadratic trajectory. Accordingly they conclude that diffusion cannot serve as a source of specific proxies for myelination. This observation might well be true also when comparing ADC and T1 relaxation with larger lifespan cohorts as reported Yeatman et al [27], who noticed different age related behaviors of ADC and T1 relaxtion, hence must reflect different biological processes according to them.

If we separate WM from basal ganglia, we note that the correlation between ADC and T1-relaxation time is stronger in WM and weaker in GM. Conceivably, ADC and T1-relaxation times are sensitive to certain elements of tissue microstructure which differ in WM and the deep gray nuclei. ADC, which measures the velocity of incoherent water motion, is dependent not only on volume of intra- and extra-cellular space but also on the tortuosity of the micro-environment and the level of myelination, whereas T1-relaxation time is only sensitive to the macromolecular concentration which is mainly related to the amount of myelination and tissue hindrance [47,48].

Mapping with T1-relaxation time using MP2RAGE sequence in normal patients may be used to track delays in myelination in neurological diseases some of which cannot be classified by clinicians. And this study comes as a complement to larger studies [24–26] but with a clinical perspective where T1 mapping can only be conceived within a clinical protocol where additional imaging must be done in the same session and within a limited time slot. In addition, several comparative studies using normalized ADC maps have already demonstrated the value of ADC as a marker of abnormal cerebral development in tuberous sclerosis, leukodystrophy, peroxisomal disorders, Krabbe disease, Canavan disease, metachromatic leukodystrophy and mitochondriopathies [7,49].

Our study collected data retrospectively on a limited total number of patients. Although patients suffering from a significant medical condition were excluded, patients were investigated for clinical symptoms such as headache or seizure which initially warranted MR imaging. A prospective study on carefully selected healthy volunteers may differ from our study. However, given the inter-subject variability and other potential confounding bias when recruiting healthy volunteers, such as socio-education status, ethnicity, gender, we do not expect to observe significantly different results. Additionally, the cross-sectional design limits longitudinal inferences on individual neurodevelopment. From a technical standpoint, three different 3.0 Tesla devices were used. Although technical parameters of the MP2RAGE sequence were identical, reproducibility on all three devices was not tested. However this sequence and the related T1 maps are FDA approved ensuring good reliability (Supplementary material S2 File provides a validation study performed by the vendor). We took a simple circular ROI approach instead of segmenting with great detail or with the help of tractography major fasciculi in the brain. Our analysis are potentially more difficult in interpret in terms of neurobiology as compared to other studies (e.g. [27] but has the advantage to be readily transferable into the clinical reading room. ROIs were manually traced leading to some inter-observer variability and erroneous measurements of caudate nucleus and the corpus callosum because of partial volume averaging.

## Conclusion

T1 relaxation time is a useful marker of brain late maturation, which can be measured reliably on high resolution images in short scan time during a routine clinical study. The age dependent tables provide normative data which may be used in a clinical setting. We confirm that T1-relaxation time and ADC evolve differentially depending of brain region during 1 and 20 years of age reflecting differential brain maturation trajectories. T1 and ADC markers do not significantly differ in their ability to reflect brain maturation but their intrinsic difference in bio-physical sensitivity make them complementary rather than redundant tools.

## Supporting information

S1 File MP2RAGE morpho skyra. (PDF)

S2 File Validation measurement of MP2RAGE sequence extension. (DOC)

S3 File Statistical analysis. (XLSX)

S1 Table Original data. (XLSX)
