# Supplementary material for: Rapid high resolution T1 mapping as a marker of brain development: Normative ranges in key regions of interest
Source: PLoS One. 2018 Jun 14;13(6):e0198250. doi: 10.1371/journal.pone.0198250 (PMC6002025; doi:10.1371/journal.pone.0198250)
Supplement: S1 File — (PDF) [file pone.0198250.s001.pdf]

Table of contents

\\Skyra
Sk_Neuro
A Cerveau
Sequences T1
mp2rage_morpho_wip900D

\\Skyra\Sk_Neuro\A Cerveau\Sequences T1\mp2rage_morpho_wip900D
TA: 8:22 PM: REF Voxel size: 1.0×1.0×1.2 mmPAT: 3 Rel. SNR: 1.00 : tfl

Properties

Prio recon	Off
Load images to viewer	On
Inline movie	Off
Auto store images	On
Load images to stamp segments	Off
Load images to graphic segments	Off
Auto open inline display	Off
Auto close inline display	Off
Start measurement without further preparation	Off
Wait for user to start	Off
Start measurements	Single measurement

Routine

Slab group	1
Slabs	1
Dist. factor	50 %
Position	Isocenter
Orientation	Sagittal
Phase enc. dir.	A >> P
AutoAlign	Head > Basis
Phase oversampling	0 %
Slice oversampling	0.0 %
Slices per slab	176
FoV read	256 mm
FoV phase	93.8 %
Slice thickness	1.20 mm
TR	5000.0 ms
TE	2.9 ms
Averages	1
Concatenations	1
Filter	Distortion Corr.(3D), Prescan Normalize
Coil elements	HE1-4

Contrast - Common

TR	5000.0 ms
TE	2.9 ms
Magn. preparation	Non-sel. IR
TI 1	700 ms
TI 2	2500 ms
Flip angle 1	4 deg
Flip angle 2	5 deg
Fat suppr.	None
Water suppr.	None

Contrast - Dynamic

Averages	1
Averaging mode	Long term
Reconstruction	Magnitude
Measurements	1
Multiple series	Each measurement

Resolution - Common

FoV read	256 mm
FoV phase	93.8 %
Slice thickness	1.20 mm
Base resolution	256
Phase resolution	100 %
Slice resolution	100 %

Resolution - Common

Phase partial Fourier	Off
Slice partial Fourier	Off

Resolution - iPAT

PAT mode	GRAPPA
Accel. factor PE	3
Ref. lines PE	32
Accel. factor 3D	1
Reference scan mode	Integrated

Resolution - Filter Image

Image Filter	Off
Distortion Corr.	On
Mode	3D
Unfiltered images	Off
Prescan Normalize	On
Unfiltered images	Off

Resolution - Filter Rawdata

Raw filter	Off
Elliptical filter	Off

Geometry - Common

Slab group	1
Slabs	1
Dist. factor	50 %
Position	Isocenter
Orientation	Sagittal
Phase enc. dir.	A >> P
Slice oversampling	0.0 %
Slices per slab	176
FoV read	256 mm
FoV phase	93.8 %
Slice thickness	1.20 mm
TR	5000.0 ms
Multi-slice mode	Single shot
Series	Interleaved
Concatenations	1

Geometry - AutoAlign

Slab group	1
Position	Isocenter
Orientation	Sagittal
Phase enc. dir.	A >> P
AutoAlign	Head > Basis
Initial Position	Isocenter
L	0.0 mm
P	0.0 mm
H	0.0 mm
Initial Rotation	0.00 deg
Initial Orientation	Sagittal

Geometry - Navigator

Geometry - Tim Planning Suite

Set-n-Go Protocol	Off
Table position	H
Table position	0 mm
Inline Composing	Off

System - Miscellaneous

Positioning mode	REF
Table position	H
Table position	0 mm
MSMA	S - C - T
Sagittal	R >> L
Coronal	A >> P
Transversal	F >> H
Coil Combine Mode	Adaptive Combine
Save uncombined	Off
Matrix Optimization	Off
AutoAlign	Head > Basis
Coil Select Mode	Default

System - Adjustments

B0 Shim mode	Standard
B1 Shim mode	TrueForm
Adjust with body coil	Off
Confirm freq. adjustment	Off
Assume Dominant Fat	Off
Assume Silicone	Off
Adjustment Tolerance	Auto

System - Adjust Volume

Position	Isocenter
Orientation	Sagittal
Rotation	0.00 deg
A >> P	240 mm
F >> H	256 mm
R >> L	212 mm
Reset	Off

System - pTx Volumes

B1 Shim mode	TrueForm
Excitation	Non-sel.

System - Tx/Rx

Frequency 1H	123.221863 MHz
Correction factor	1
Gain	Low
Img. Scale Cor.	1.000
Reset	Off
? Ref. amplitude 1H	0.000 V

Physio - Signal1

1st Signal/Mode	None
TR	5000.0 ms
Concatenations	1

Physio - Cardiac

Magn. preparation	Non-sel. IR
TI 1	700 ms
TI 2	2500 ms
Fat suppr.	None
Dark blood	Off
FoV read	256 mm
FoV phase	93.8 %
Phase resolution	100 %

Physio - PACE

Resp. control	Off
Concatenations	1

Inline - Common

Subtract	Off
Measurements	1
StdDev	Off
Save original images	On

Inline - MIP

MIP-Sag	Off
MIP-Cor	Off
MIP-Tra	Off
MIP-Time	Off
Save original images	On

Inline - Composing

Inline Composing	Off
Distortion Corr.	On
Mode	3D
Unfiltered images	Off

Inline - MapIt

Save original images	On
MapIt	T1 map
Flip angle 1	4 deg
Flip angle 2	5 deg
Measurements	1
Contrasts	1
TR	5000.0 ms
TE	2.9 ms

Sequence - Part 1

Introduction	On
Dimension	3D
Elliptical scanning	Off
Reordering	Linear
Asymmetric echo	Off
Contrasts	1
Flow comp.	No
Multi-slice mode	Single shot
Echo spacing	6.9 ms
Bandwidth	240 Hz/Px

Sequence - Part 2

RF pulse type	Fast
Gradient mode	Fast
Excitation	Non-sel.
RF spoiling	On
Incr. Gradient spoiling	Off
Turbo factor	176

Sequence - Special

Morphometry Analysis	On
Skull Stripping	Off
Label Map	On
Deviation Map	On
Disease Class.	On
XML Export	Off
Uniform Image	On
Denoise Weighting	100
Complex Div. Image	Off
Synthetic TI 0	0 ms
Synthetic TI 1	0 ms
New AC Mode	Off
IcePat	Off
Iter. Denoising	Off
FLAWS	OFF

Sequence - Special

FID MoCo Logging	Off
FID Phase Logging	Off
FID Phase Correct	Off
Ext. Inversion Pulse	Off

Sequence - Assistant

Mode	Off
