# Supplementary material for: Rapid high resolution T1 mapping as a marker of brain development: Normative ranges in key regions of interest
Source: PLoS One. 2018 Jun 14;13(6):e0198250. doi: 10.1371/journal.pone.0198250 (PMC6002025; doi:10.1371/journal.pone.0198250)
Supplement: S2 File — (DOC) [file pone.0198250.s002.doc]

**Validation Measurement of MP2RAGE Sequence Extension**

**Goal of Validation Measurement**

Uniform images (UNI) and T1 maps as obtained by the newly introduced “MP2RAGE” extension of the standard TurboFLASH sequence are to be validated. To this end, a T1 phantom, i.e. a phantom comprising different compartments which contain liquids with different T1 values, was employed to validate both the T1 contrasts in the UNI images as well as the quantitative T1 values obtained from the T1 maps.

The T1 maps are expected to reflect the real T1 values within a ±10 % margin. The MP2RAGE T1 mapping technique is optimised for a T1 value range between 400 ms and 1800 ms. Thus, T1 values outside this range shall not be considered.

**Material & Methods**

The T1/T2 test phantom „Eurospin II“ by Diagnostic Sonar Ltd., Livingston, Scotland, UK was used for T1 contrast and mapping validation. It was placed in the standard head coil of the respective scanner. The phantom contains 14 tubes with differently doped liquids confined in a water-filled cylinder. The numbered tubes are arranged as follows (cf. also transverse image slices):

*anterior*

                         #3        #5

                 #6     #7        #8    #9

*right*                                                 *left*

                #11    #12     #13    #14

                         #15    #16

*posterior*

The T1 values corresponding to the different tubes are reported in Table 1.

| **No. Tube** | **T1 @ 296 K 1.5T (3T)** | **No. Tube** | **T1 @ 296 K 1.5T (3T)** |
| --- | --- | --- | --- |
| 3 | 329 ms (331 ms) | 11 | 992 ms (1007 ms) |
| 5 | 496 ms (502 ms) | 12 | 1603 ms (1615 ms) |
| 6 | 492 ms (496 ms) | 13 | 1068 ms (1078 ms) |
| 7 | 664 ms (674 ms) | 14 | 1137 ms (1153 ms) |
| 8 | 659 ms (666 ms) | 15 | 1278 ms (1293 ms) |
| 9 | 828 ms (841 ms) | 16 | 1403 ms (1422 ms) |

*Table 1 - T1 values of test phantom at 1.5 and 3 Tesla and room temperature (23°C) taken from the phantom’s manual*

Measurements were performed on a Siemens MAGNETOM Aera (1.5T) and a Siemens MAGNETOM Skyra (3T) scanner employing the respective standard protocols which are delivered with the MP2RAGE sequence extension. The essential sequence parameters can be seen in Table 2. The measurement protocol comprised four scans acquired in the scanner’s isocentre. The first three scans were in sagittal (SAG), transversal (TRA) and coronal (COR) orientation, the last one again in sagittal orientation in order to assess intra-scan variability.

| **Sequence Parameter** | **1.5 T Protocol** | **3 T Protocol** |
| --- | --- | --- |
| Acquisition Mode | 3D | 3D |
| Matrix Size | 192 x 192 | 256 x 256 |
| No. Partitions | 160 | 176 |
| Resolution | 1.3 x 1.3 x 1.2 mm^3^ | 1 mm isotropic |
| Inversion Time 1 (TI1) | 600 ms | 700 ms |
| Inversion Time 2 (TI2) | 2000 ms | 2200 ms |
| Flip Angle 1 | 6 deg | 4 deg |
| Flip Angle 2 | 5 deg | 5 deg |
| Repetition Time | 5 s | 5 s |
| Parallel Acquisition Factor | 2 | 3 |

*Table 2 - Essential sequence parameters in the 1.5 T and 3 T measurement protocols*

A temperature of 22° C (21° C) was measured at the Aera (Skyra) scanner at the beginning of the measurement. After the phantom was positioned, it was given 5 minutes to acclimatise.

The transversely acquired set of T1 maps at both field strengths was analysed drawing 12 regions-of-interest (ROI) encompassing all vials in the phantom. The ROIs had a size of 248 (405) pixels at 1.5 T (3T). The size differed because of the different resolutions at the two field strengths.

To assess intra-scan variability, difference images of the UNI images and T1 maps of the two acquired sagittal scans were calculated for both field strengths. ROIs were drawn in the slice centre to evaluate the difference image intensity range.


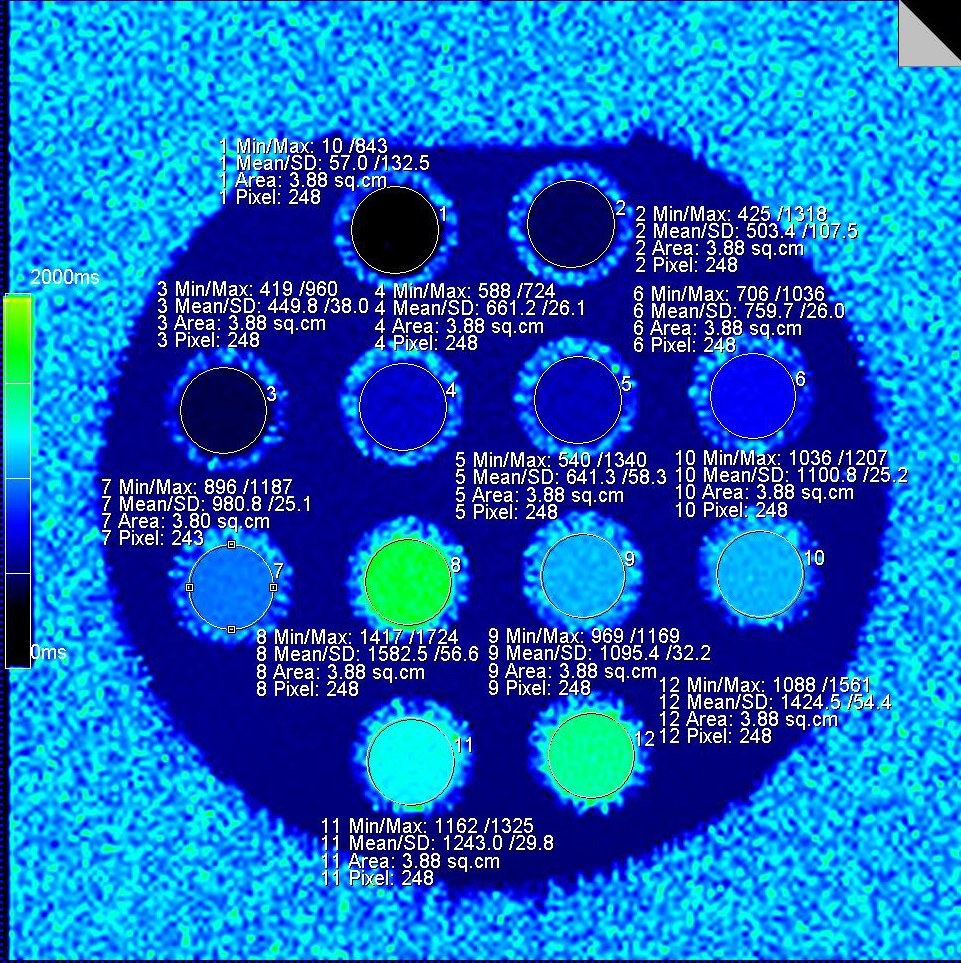

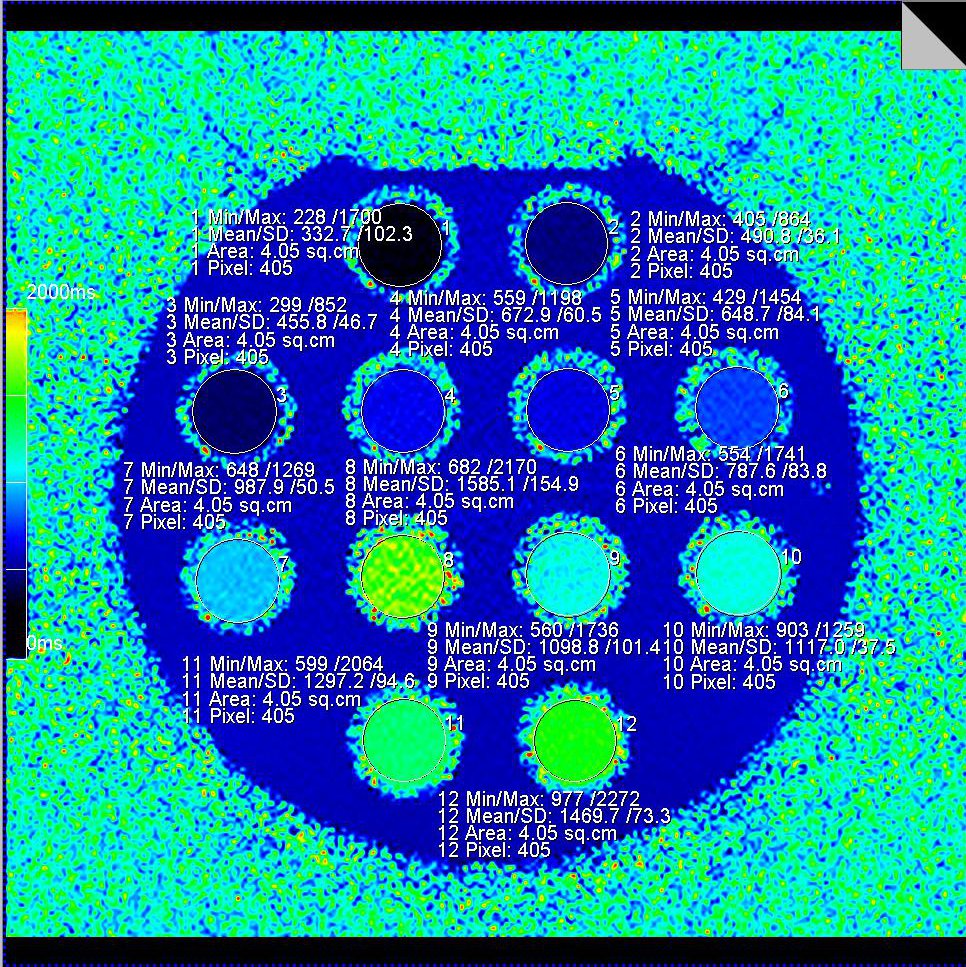


*Figure 1 - T1 ROI measurements at 1.5 T (left) and 3T (right)*

**Results**

Mean T1 values of the analysed ROIs are reported in Table 3 and Figure 1.

| **No. Tube** | **1.5T nom. [ms]** | **1.5T meas.**  **[ms]** | **Diff. [ms]** | **Diff. [%]** | **3T nom. [ms]** | **3T meas. [ms]** | **Diff. [ms]** | **Diff. [%]** |
| --- | --- | --- | --- | --- | --- | --- | --- | --- |
| 3 | 329 | 57 | 272 | -82.7% | 331 | 333 | 2 | 0.6% |
| 5 | 496 | 503 | -7 | 1.4% | 502 | 491 | -11 | -2.2% |
| 6 | 492 | 450 | 42 | -8.5% | 496 | 456 | -40 | -8.1% |
| 7 | 664 | 661 | 3 | -0.5% | 674 | 673 | -1 | -0.1% |
| 8 | 659 | 641 | 18 | -2.7% | 666 | 649 | -17 | -2.6% |
| 9 | 828 | 760 | 68 | -8.2% | 841 | 788 | -53 | -6.3% |
| 11 | 992 | 980 | 12 | -1.2% | 1007 | 988 | -19 | -1.9% |
| 12 | 1603 | 1583 | 20 | -1.2% | 1615 | 1585 | -30 | -1.9% |
| 13 | 1068 | 1095 | -27 | 2.5% | 1078 | 1099 | 21 | 1.9% |
| 14 | 1137 | 1101 | 36 | -3.2% | 1153 | 1117 | -36 | -3.1% |
| 15 | 1278 | 1243 | 35 | -2.7% | 1293 | 1297 | 4 | 0.3% |
| 16 | 1403 | 1425 | -22 | 1.6% | 1422 | 1470 | 48 | 3.4% |

*Table 3 - Nominal and measured T1 values at 1.5 and 3 T*

Tube no. 3 with a nominal T1 of ca. 330 ms does not fall in the previously defined value range of biological T1 values which can be consistently measured by the MP2RAGE technique. This can be clearly seen by the difference to the measured value at 1.5 T. At 3T, however, a precise measurement is still possible. The reason for this behavior is the large slope of the curve mapping MP2RAGE uniform image intensities to T1 values outside the T1 value range of about 400ms-1800ms, yielding increasing errors for small changes in the uniform intensities. Notably, the 1.5T protocol differs from the 3T protocol, slightly shifting the interval of large slopes, which in turn leads to the precision difference measuring tube 3.

Overall, the T1 measurement analysis show good to excellent agreement with the nominal values. The largest differences were observed in vials no. 6 and no. 9 with about 8%. All other values are show a per cent difference of 3.4% or better. Notably, the measurements at the two field strengths differed by maximally 3.6% corrected for the real T1 difference of the phantom (not considering tube #3).

The uniform (UNI) images show contrast differences depending on different T1 values in the tubes as expected.

A representative slice of the difference images from the two sagittal acquisitions of both the difference UNI image and the T1 map can be seen in Figure 2 . Residual intensities and T1 differences reached no significant level.


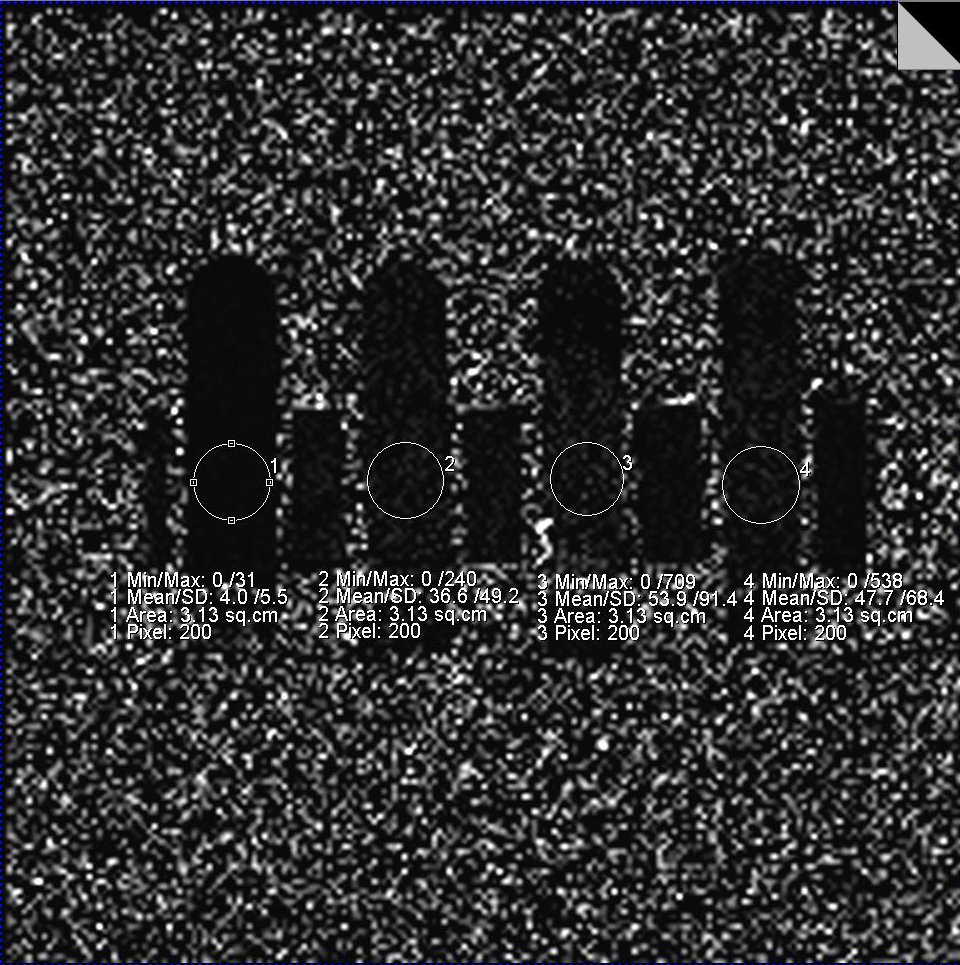

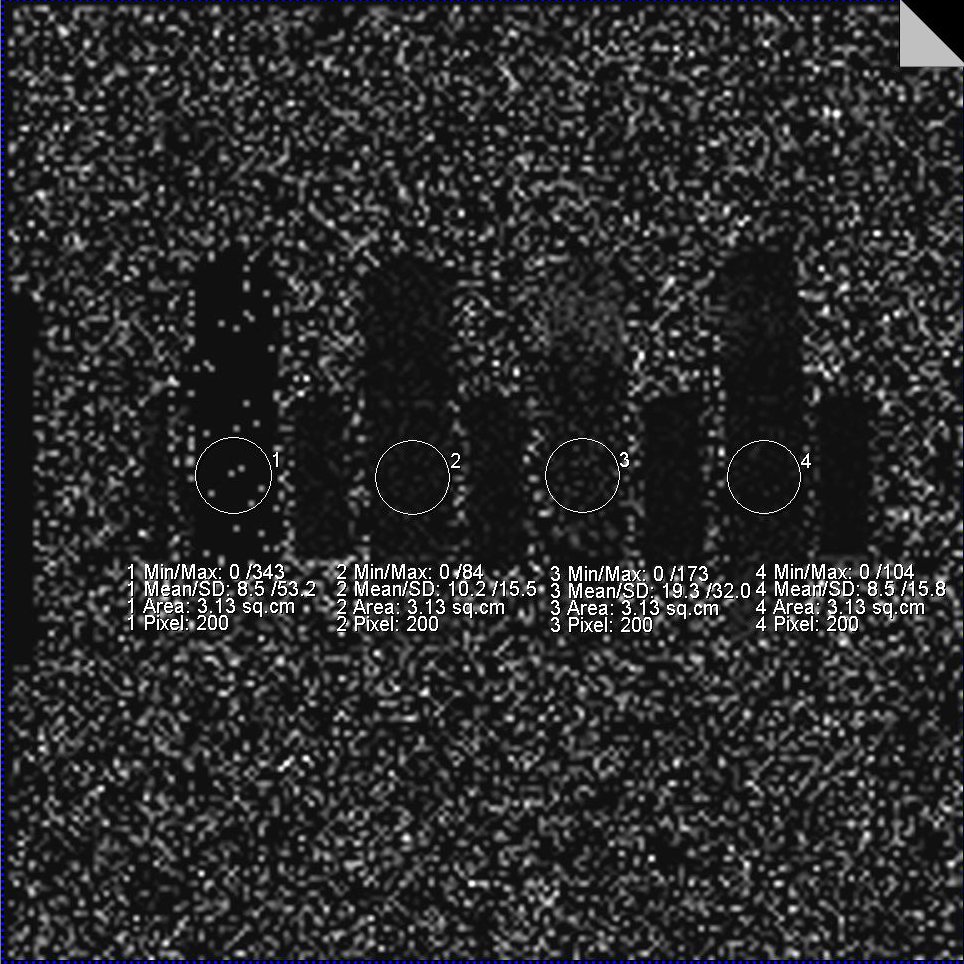


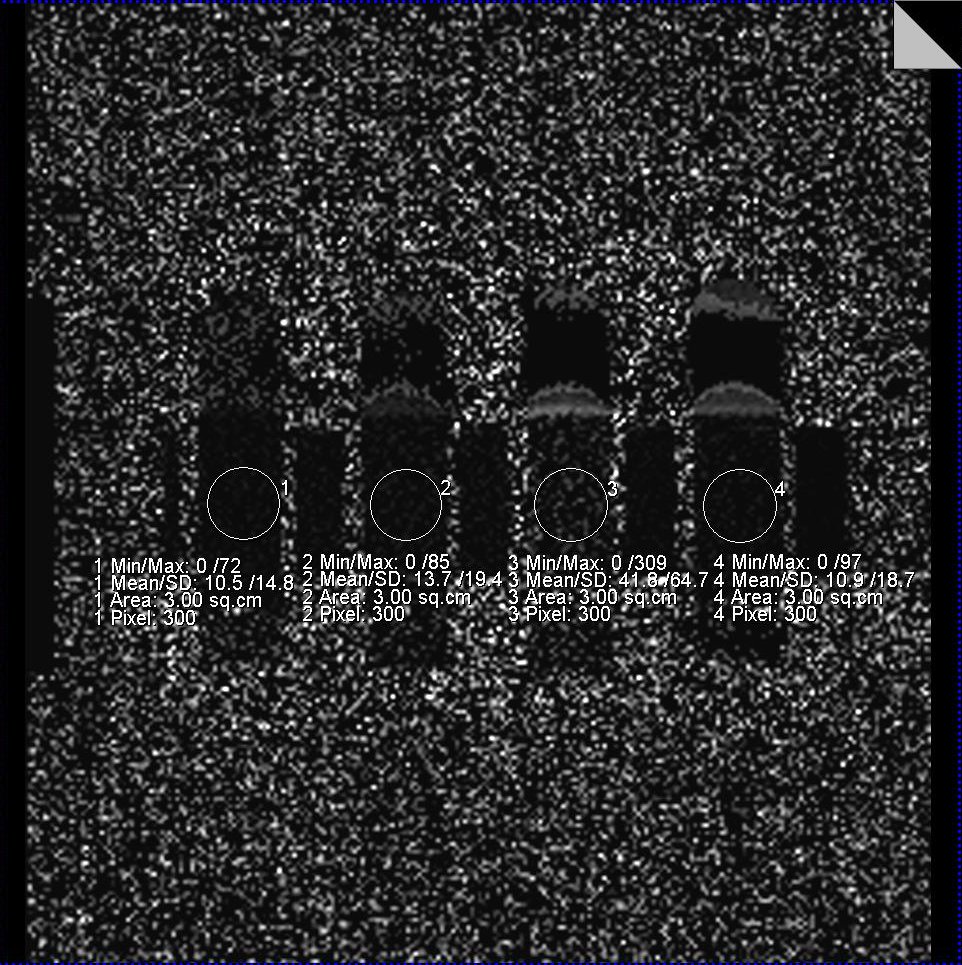

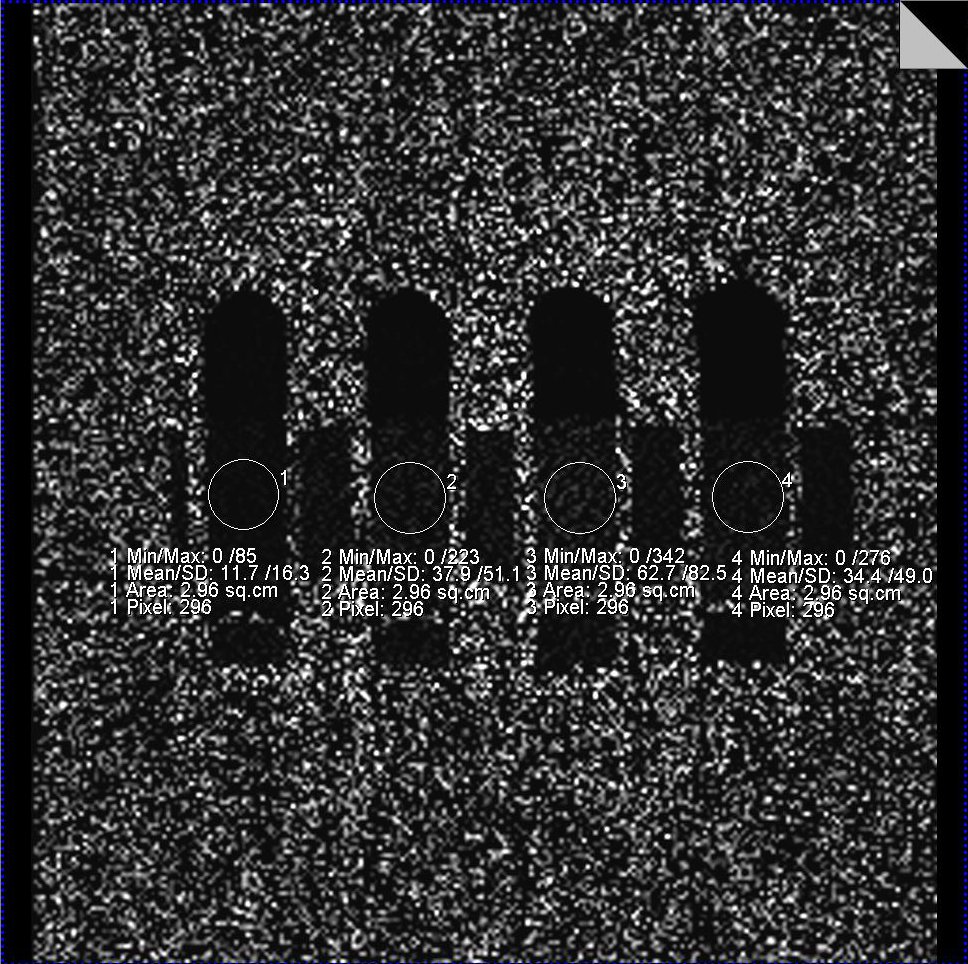


*Figure 2 – Representative difference image slice of UNI (left) and T1 map (right)
at 1.5 Tesla (top) and 3 Tesla (bottom).*

**Conclusion**

The obtained results of the MP2RAGE measurements are in good agreement with the real values for the defined biological value range. Also, quantitative results obtained at the two different field strengths showed good to excellent agreement. The difference images revealed intra-scan comparability of both UNI images and T1 maps with high accuracy.
